# Bio-Inspired Eco-Friendly Superhydrophilic/Underwater Superoleophobic Cotton for Oil-Water Separation and Removal of Heavy Metals

**DOI:** 10.3390/biomimetics7040177

**Published:** 2022-10-26

**Authors:** Feiran Li, Jian Wang, Zhuochao Wang, Dongchao Ji, Shuai Wang, Pengcheng Wei, Wenxin Cao

**Affiliations:** 1 Key Laboratory of Micro-Systems and Micro-Structures Manufacturing (Ministry of Education), School of Mechatronics Engineering, Harbin Institute of Technology Xidazhi, No. 92, Harbin 150001, China; 2 School of Mechatronics Engineering, Heilongjiang East University, Harbin 150066, China; 3 Center for Composite Materials and Structures, Harbin Institute of Technology, Harbin 150080, China

**Keywords:** superhydrophilic/underwater superoleophobic, oil-water separation, emulsions, chitosan, water purification

## Abstract

Effective integrated methods for oil-water separation and water remediation have signifi-cance in both energy and environment fields. Materials with both superlyophobic and superlyophilic properties toward water and oil have aroused great attention due to their energy-saving and high-efficient advantages in oil-water separation. However, in order to fulfill the superlyophobicity, low surface tension fluorinated components are always being introduced. These constituents are environmentally harmful, which may lead to additional contamination during the separating process. Moreover, the heavy metal ions, which are water-soluble and highly toxic, are always contained in the oil-water mixtures created during industrial production. Therefore, material that is integrated by both capacities of oil-water separation and removal of heavy metal contamination would be of significance in both industrial applications and environmental sustainability. Herein, inspired by the composition and wettability of the shrimp shell, an eco-friendly chitosan-coated (CTS) cotton was developed. The treated cotton exhibits the superhydrophilic/underwater superoleophobic property and is capable of separating both immiscible oil-water mixtures and stabilized oil-in-water emulsions. More significantly, various harmful water-soluble heavy metal ions can also be effectively removed during the separation of emulsions. The developed CTS coated cotton demonstrates an attractive perspective toward oil-water separation and wastewater treatment in various applications.

## 1. Introduction

Pollutions caused by industry-induced heavy metal-contaminated oil-water mixtures have become a pressing issue in areas such as marine conservation, manufacture and resource recovery [1,2,3,4]. Effective integrated methods for oil-water separation and water remediation have significance in both energy and environment fields.

For oil-water separation, traditional methods, such as hydrocyclone and sedimentation, which are generally used for immiscible oil-water mixtures separation, are either energy-intensive or time-consuming [5,6]. For more complex oily wastewater and emulsions, electrochemical and biological treatments are commonly used. These methods, however, are inefficient or have high energy consumption [1,7]. Since the early 2000s, materials with both superlyophobic (liquid contact angle greater than 150° and tilt angle smaller than 10°) and superlyophilic (liquid contact angle smaller than 10°) properties toward water and oil have aroused great attention due to their energy-saving and high-efficient advantages in oil-water separation and wastewater treatment. These materials are generally divided into two types: superhydrophobic/superoleophilic (oil removing) and superoleophobic/superhydrophilic (water removing) material [8,9,10,11,12]. For the separation of oil-water mixtures, these materials are usually attached to porous substrates, the affiliative phase wets and penetrates the material, while the repellent phase is rejected by the material. Superhydrophobic/superoleophilic material is the first developed selective superwetting material used for oil-water separation. These materials preserve water repellent and oil-wetting behavior [13,14,15,16,17,18], whereas materials with superhydrophobic/superoleophilic properties are easily contaminated or even blocked by viscous oils due to their oil affinity, which may lead to unexpected interruption or invalidation during oil-water separation. In contrast, materials with oil-repellent and water-wetting behavior are more suitable for oil-water separation. Several methods have been developed to fabricate superoleophobic/superhydrophilic materials and have proved themselves as competitive solutions in the application of oil-water separation [19,20,21,22,23,24,25]. However, low surface energy is the key factor in constructing superlyophobic surfaces [1,21]. In order to get lower surface tension, fluorinated components are always introduced in fabricating superhydrophobic and superoleophobic surfaces. These fluorinated constituents are environmentally harmful, which may lead to additional contamination during the separating process. Thus, eco-friendly material with oil-repellent and water-wetting behavior is of interest in practical applications of oil-water separation.

Inspired by the underwater oil-repellent property of the fish scale, Xue et al. proposed a concept in fabricating material with superhydrophilic and underwater superoleophobic properties [26]. Unlike other superlyophobic materials on which a prerequisite low surface energy component is required, materials with superhydrophilic and underwater superoleophobic properties usually preserve high surface tension in the air environment, which provides a feasible solution for the fabrication of non-toxic selective superwetting material. Following this strategy, materials with superhydrophilic/underwater superoleophobic properties have been developed by various methods and were used to separate oil-water mixtures [27,28,29]. Mesh or fabric is used as substrates for separating immiscible oil-water mixtures. These mesh/fabric-based superhydrophilic/underwater superoleophobic structures, however, failed to separate more complex oil-in-water emulsions in which microdroplets (normally smaller than 20 µm) of oil dispersed in water because the microdroplets can easily pass through the substrates [6]. Thus, superhydrophilic/underwater superoleophobic material, which can separate both immiscible oil-water mixtures and emulsions, is of interest in various applications.

Apart from the flexibility in separating different types of oil-water mixtures, the ability of water remediation during oil-water separation is of great significance. Oil-water mixtures created in practice are so complicated that a single separation process cannot satisfy the requirements for wastewater treatment. The situation is especially severe for industrial waste oil-water mixtures in which highly toxic heavy metal ions are dissolved [30]. Traditional methods for removal of heavy metal ions from aqueous solutions, such as ion exchange and electrodialysis, need additional treating process, which would reduce the efficiency of wastewater treatment and increase the cost of equipment [31]. Therefore, material which is integrated by both capacities of oil-water separation and removal of heavy metal contamination would be of significance in both industrial applications and environmental sustainability.

**Figure 1:**
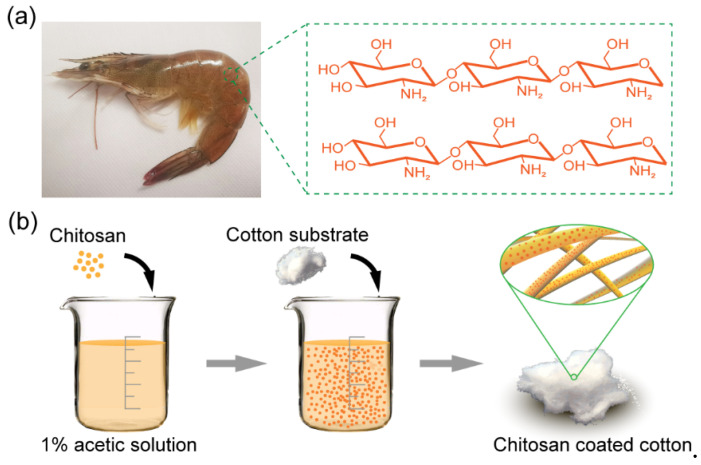
(**a**) The optical image of the shrimp and the chemical structure of the chitosan. (**b**) The fabrication process of the CTS-coated cotton.

Chitosan (CTS), a non-toxic polymer which is synthesized from the main component of the crustacean shells, is considered as one of the most effective absorbents in wastewater treatment due to its chelation capacity toward heavy metal ions. The shrimp shell, which is a typical example of the crustacean shell, also preserves excellent anti-fouling behavior due to its underwater superoleophobic property (Figure 1a) [32,33,34,35,36]. Taking full advantage of the properties of CTS, fabricating CTS-based materials, which can remove heavy metal ions during oil-water separation, can have great significance in intricate liquid treatment. Unfortunately, integrated materials of this kind have rarely been reported. Since sufficient contact of solution and chitosan (the adsorbents) is essential for removing metal ions. In general, the ion contaminants are removed by mixing and filtrating methods [37,38,39]. In recent years, superhydrophilic/underwater superoleophobic CTS-based porous materials have been developed [40,41,42,43]. Most of these materials preserve excellent performance in separating immiscible oil-water mixtures. However, these materials lack the metal ions-removing capacities because of the insufficient contact of the CTS and the aqueous solution. Hence, fabricating CTS-based materials with both superhydrophilic/underwater superoleophobic property for effective oil-water separation and sufficient liquid contact efficiency is still challenging.

In this study, an eco-friendly superhydrophilic/underwater superoleophobic CTS-modified cotton was developed by a facile method. Benefiting from the multitudinous fibrous structure and highly compressibility of the cotton substrate, the CTS-coated cotton can not only separate immiscible oil-water mixtures but also stabilized oil-in-water emulsions with a considerable separation capacity. Most noticeably, due to the sufficient contact of the liquid and the multitudinous fibers on the treated cotton, the harmful water-soluble heavy metal ions, such as bivalent cuprum (Cu^2+^), trivalent iron (Fe^3+^), univalent silver (Ag^1+^) and hexavalent chromium (Cr^6+^), can also be effectively removed during the separation of emulsions. This eco-friendly and effective CTS-coated cotton demonstrates an attractive perspective toward oil-water separation and wastewater treatment in various fields.

## 2. Materials and Methods

### 2.1. Materials

Chitosan (deacetylation ≥ 95%, viscosity~100–200 mpa.s), acetate acid (ACS, ≥99.7%), cotton, hexadecane (GC, >99.5%) and 1,2-dichloroethane (GC, >99.8%) were achieved from Aladdin (Shanghai, China). Diiodomethane (99%, ReagentPlus^®®^) was purchased from Sigma Aldrich. TWEEN 60 was obtained from Shanghai Macklin Bio-Chem Corporation (Shanghai, China). Diesel oil was obtained from the Sinopec gas station (Harbin, China), soybean oil (Non-GMO, Jiusan Group) was brought from Carrefour Market (Harbin, China). Potassium dichromate (K_2_Cr_2_O_7_, MW 294.18, ≥99.0%), Copper(II) chloride dehydrate (CuCl_2_•2H_2_O, MW 170.48) and ferric chloride (FeCl_3_, MW 162.20, 97%) were purchased from Fengchuan Chem. (Tianjin, China). All chemicals were used as received. The parameters of the liquid used in this study are exhibited in Table 1.

### 2.2. Preparation of CTS-Coated Cotton

#### 2.2.1. Pretreatment of the Cotton

A piece of pristine cotton (~0.5 g) was ultrasonically washed with ethanol solution 2 to 3 times, and immersed into a beaker containing 2% NaOH aqueous solution and followed by 10 min of magnetic stirring. Then, the cotton was washed several times with deionized water until the pH level of the filtrate reached neutrality. Finally, the pre-treated cotton was dried at 60 °C for 1 h.

#### 2.2.2. Preparation of CTS-Coated Cotton

The preparation process is shown in Figure 1b: 2.0 g chitosan was initially dissolved in 50 mL 1% (w%) acetic solution and magnetically stirred for 1 h to make the chitosan coating; then a piece of prewashed cotton was immersed into the coating solution and ultrasonic shake for 2 h before drying at 80 °C for 3 h; the treated cotton was finally washed with hot water (~50 °C) several times and then dried at 80 °C before use.

### 2.3. Oil-Water Separation

#### 2.3.1. Separation of Immiscible Oil-Water Mixtures

To separate immiscible oil-water mixtures, 0.5 g of treated cotton was firstly flattened by a polyfluortetraethylene plate. The flattened cotton was then sandwiched between a glass tube and a suction flask. The gaps between the tubes were then sealed with silicone sealant. The combination was then set above the beaker with the cotton side up. The cotton was totally wetted by water before separation. Immiscible oil-water mixtures (volume ratio between water and the corresponding oil is 1:1) was poured onto the CTS-coated cotton. For the separation test in the harsh environment, the pH of water was pre-adjusted by hydrochloric acid (HCL) and NaOH before mixing with oil liquids.

#### 2.3.2. Separation of Heavy Metal Ion-Contaminated Emulsions

The of metal ion-contaminated oil-in-water emulsions were prepared by mixing the metal ion-contaminated aqueous solution with an emulsifier and the corresponding oil. Fe^3+^, Cu^2+^ and Cr^6+^ were selected as target pollutants. Diesel, soybean oil and hexadecane were selected as oils. In detail, 20 mg L^−1^ of the corresponding ion-contaminated aqueous solution was obtained by diluting a standard stock solution (2.0 g/L). TWEEN 60, the corresponding oil and metal ion-contaminated aqueous solution were mixed in a ratio of 1:10:100 by volume. Each mixture was vibrated with an ultrasonic homogenizer (Xinzhi JY92-11N, 20 kHz frequency) for 3 h to form an emulsion.

For the emulsion separation, a piece of chitosan-coated cotton (~1 g) was pushed into an injector barrel as tightly as possible, the density of the compressed cotton was about 0.28 g cm^−3^. In order to prewet the CTS-coated cotton, 5 mL of deionized water was firstly added into the barrel, then the oil-in-water emulsion was poured into a barrel, followed by a gravity-driven continuous separation of 30 mL test sample.

### 2.4. Characterization and Measurement

The morphology characterization was performed by the laser scanning confocal microscope (Zeiss LSM700) and Scanning Electron Microscopy (SEM, Carl Zeiss Jena, Germany) at a 10 kV acceleration voltage at a working distance of 8 mm. The chemical constituents were detected by Fourier Transform infrared spectrophotometer (FT-IR, Nicolet iS50, Thermo Scientific). The contact angles were measured by the optical contact angle meter (DropMeterTM Element A-60, Maist, Ningbo, China) at ambient environment conditions and each data was determined by the average value of 5 times experiments. The oil distributions in the feeds and filtrates were characterized by Leica DVM6s 3D Microscope (Leica, Germany). The oil content in the filtrate was measured by the total organic carbon analyzer (TOC, ANALYTIKJENA Multi N/C, Jena, Germany). The metal ions in water were detected by inductively coupled plasma mass spectrometry (ICP-MS, 7800, Agilent, Santa Clara, CA, USA). For the measurement of the oil content and metal ion concentration, the data was obtained by taking an average of 5 test results with separate experiments.

The flux Q and separation efficiency *η* were calculated by Equation (1) and Equation (2), respectively.

(1) $$Q=\frac{V}{S\cdot t}$$

(2) $$\eta =\frac{C_{feed}-C_{filtrate}}{C_{feed}}\times 100\%$$

where *V* is the volume of the separated emulsion, *S* is the sectional area of the injector barrel, and *t* represents the time of the separation process. *C_feed_* and *C_filtrate_* represent the concentrations of oil in the emulsion feed and filtrate, respectively.

The removal efficiency $q_{e}$ was calculated by Equation (3):

(3) $$q_{e}=\frac{C_{0}-C_{e}}{C_{0}}\times 100\%$$

where *C*_0_ and *C*_e_ are concentrations of heavy metal ions in the feed aqueous solution in the emulsion (20 mg L^−1^) and collected filtrate after separation, respectively.

## 3. Result and Discussion

### 3.1. The Characterization of the CTS-Coated Cotton

**Figure 2:**
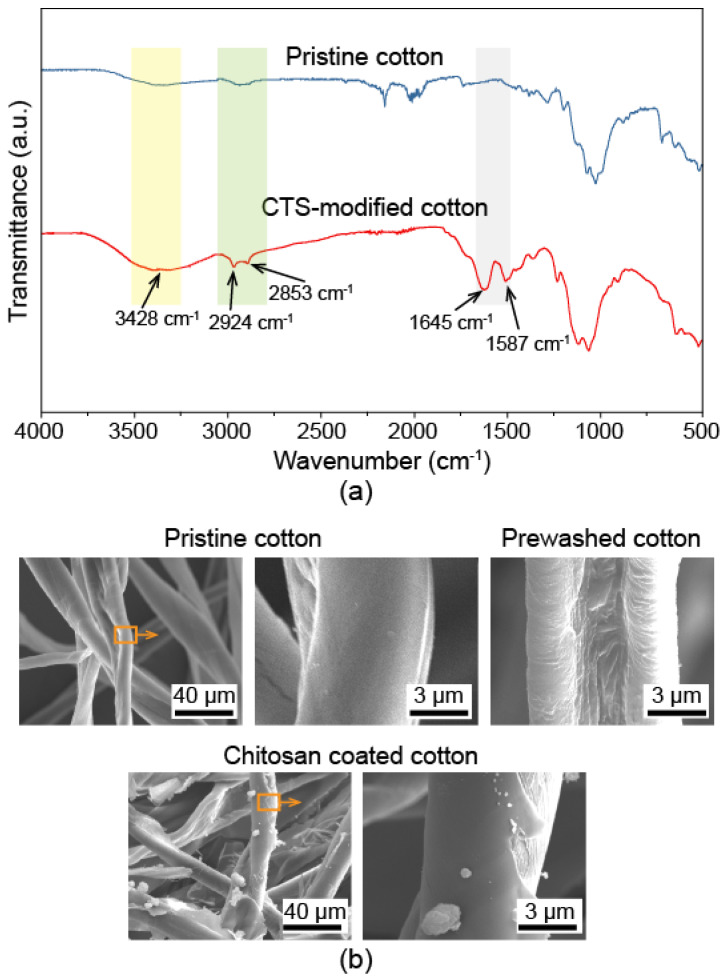
(**a**) FTIR spectra of pristine and CTS-coated cotton. (**b**) SEM morphology of pristine, pre-washed and CTS-coated cotton.

The chemical composition of the pristine and CTS-coated cotton was characterized by the Fourier transform infrared spectrophotometer (FTIR), as shown in Figure 2a. It is observed that compared with the spectra of pristine cotton, several bands were detected on CTS-coated cotton. The bands at 3428 cm^−1^ were attributed to O-H stretch vibration. The peaks at 2924 cm^−1^ and 2853 cm^−1^ corresponded to saturated alkyl groups. The peaks at 1645 cm^−1^ and 1587 cm^−1^ were attributed to N-H and amide groups, respectively [44]. These peaks demonstrate that chitosan was attached to the cotton substrate.

**Figure 3:**
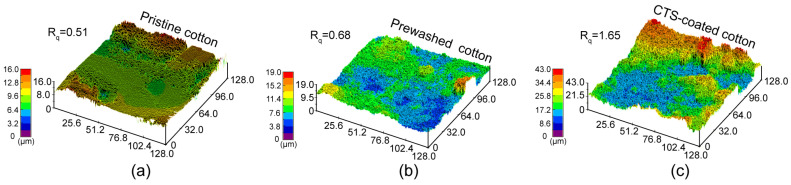
The laser scanning confocal morphology of the (**a**) pristine (**b**) prewashed cotton and (**c**) CTS-coated cotton.

The SEM morphology of the pristine cotton, prewashed cotton and CTS-coated cotton was also detected, as shown in Figure 2b. Compared with pristine cotton fiber which is smooth and flat, the prewashed cotton fiber was corrugated and wizened. The corrugation was caused by the treatment of an alkaline solution, during which the wax layer on pristine cotton was removed and led to the exposure of hydroxyl groups. The corrugation and the hydrophilic hydroxyl groups will help the CTS coating to attach better with the fibers. The cotton fibers after modification, as shown in Figure 2b, were mostly covered by CTS coating. It is observed that some agglomerated CTS particles, which were precipitated from the coating during the heating process, were also attached to cotton fibers. These particles and the coated cotton fibers constructed the hierarchal structure, which plays an important role in the construction of superwettability. To further demonstrate the change in roughness, the morphology of pristine and CTS-coated cotton was also detected by laser scanning confocal microscopy, as shown in Figure 3. The average surface roughness R_q_ on pristine cotton and pre-washed cotton were 0.51 µm and 0.68 µm, respectively. Whereas, the roughness R_q_ increased to 1.65 µm on CTS-coated cotton. Combined with the chemical composition variation and the SEM images, which are shown in Figure 2, it can be speculated that the CTS has been successfully modified on the cotton fibers. The increased roughness factor also indicates the formation of hierarchal structure, which is beneficial for enhancing the superwettability of the treated cotton.

### 3.2. The Wettability of the CTS-Coated Cotton

**Figure 4:**
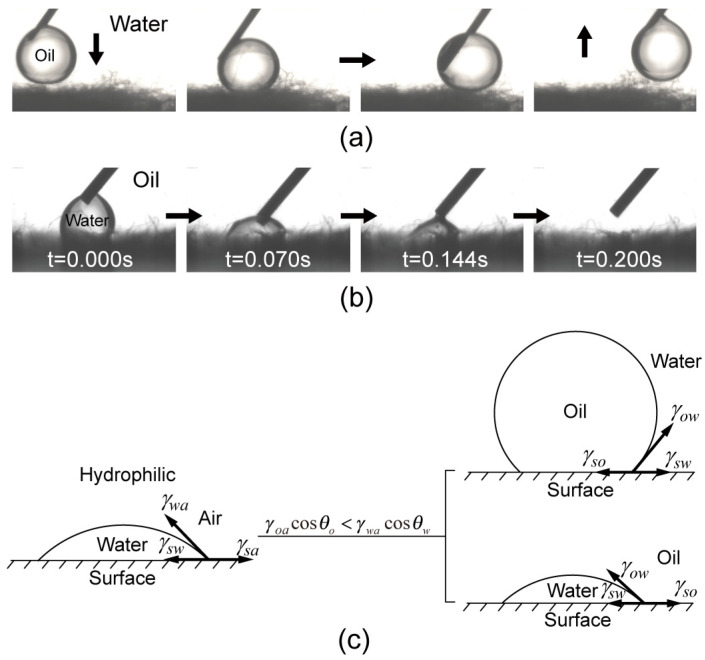
Successive frames of (**a**) the oil sliding tested in water environment and (**b**) water wetting test in oil environment on the CTS-coated cotton. Dichloroethane was selected as the test oil. (**c**) The mechanism of the underoil superhydrophilicity and underwater superoleophobicity.

The selective superwettability in the liquid environment is an essential requirement for the separation of oil-water mixtures. Therefore, the wettability of the CTS-coated cotton in a liquid environment was firstly tested, as shown in Figure 4a,b. The oil droplet can slide freely without adhesion on the CTS-coated cotton. In contrast, the water droplet in the oil environment spread and permeated rapidly into the CTS-coated cotton upon contact.

The underwater superoleophobicity and the underoil superhydrophilicity can be explained by the following Equations (4) and (5) [45,46,47]:

(4) $$\cos\theta_{ow}=\frac{\gamma_{oa}\cos\theta_{o}-\gamma_{wa}\cos\theta_{w}}{\gamma_{ow}}$$

(5) $$\cos\theta_{wo}=\frac{\gamma_{wa}\cos\theta_{w}-\gamma_{oa}\cos\theta_{o}}{\gamma_{ow}}$$

where $\theta_{ow}$ and $\theta_{wo}$ are the underwater oil contact angle and underoil water contact angle on the flat fiber surface, respectively. $\gamma_{oa}$ and $\gamma_{wa}$ are the surface tensions of oil and water, respectively. $\gamma_{ow}$ is the surface tension at the oil-water interface. Since the CTS surface is lyophilic ($\theta_{o}$ and $\theta_{w}$ are less than 90º) and the surface tensions of oil are much less than that of water. Thus, $\gamma_{oa}\cos\theta_{o}-\gamma_{wa}\cos\theta_{w}$ is negative and $\gamma_{wa}\cos\theta_{w}-\gamma_{oa}\cos\theta_{o}$ is positive. The CTS surface exhibits underwater oleophobic and underoil hydrophilic properties (Figure 4c). According to Wenzel theory, the contact angle on a rough surface can be expressed as:

(6) $$\cos\theta =R_{f}\cos\theta_{l}$$

where $\theta_{l}$ the contact angle on a flat surface, $R_{f}$ is the roughness factor which is always greater than 1 for the surface with a certain roughness. Thus, the underwater oleophobicity and underoil hydrophilicity were greatly emphasized by the hierarchical roughness created by the numerous coated fibers and resulted in the formation of underwater superoleophobicity and underoil superhydrophilicity. The consequent selective superwettability in a liquid environment enables the CTS-coated cotton to remove water from oil-water mixtures during the separation process.

**Figure 5:**
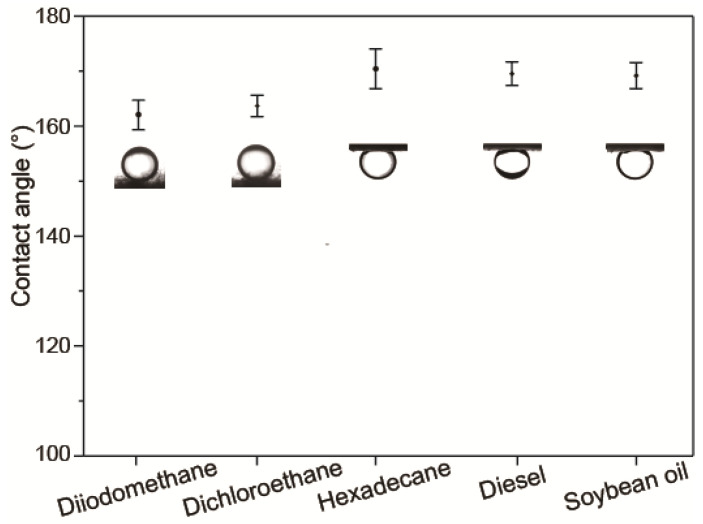
The underwater contact angles of various kinds of oils.

To further demonstrate the underwater superoleophobic property of the CTS-coated cotton, the contact angles of oils with various surface tensions and densities (Table 1) were measured, as shown in Figure 5. It is observed that the underwater contact angles of the test oils were greater than 150°, demonstrating the underwater oil-repellent behavior of the CTS-coated cotton.

### 3.3. Separation of Oil-Water Mixtures

#### 3.3.1. Separation of Immiscible Oil-Water Mixtures

**Figure 6:**
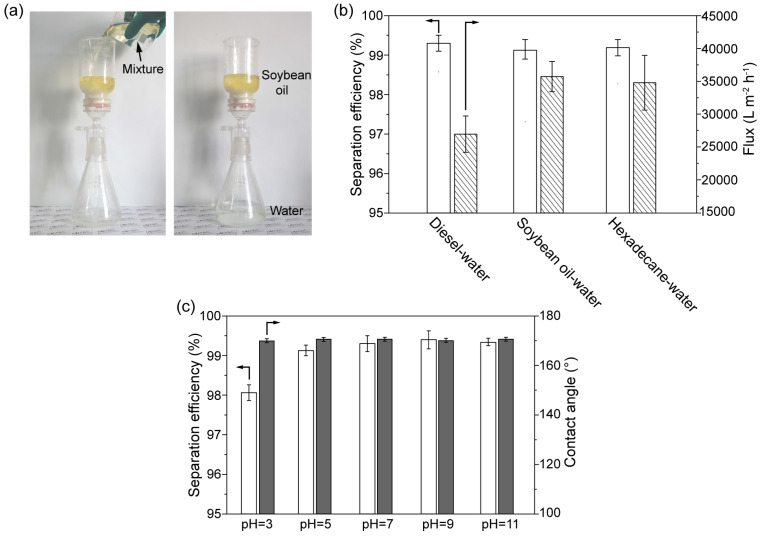
(**a**) Separation of immiscible soybean oil-water mixtures. (**b**) The separation efficiency and the flux during the separation of immiscible diesel-water, soybean oil-water and hexadecane-water mixtures, the average separation efficiencies were higher than 99% for the separation of various immiscible oil-water mixtures and the separation flux was up to 42,000 L m^−2^ h^−1^. (**c**) The separation efficiency and underwater oil contact angle in aqueous environments with pH values of pH = 3 to pH = 11. There were no obvious variation on underwater oil contact angles during variation of pH values, the oil-water separation efficiency was stable above 99% in the range of pH = 5 to pH = 11, whereas the separation efficiency decreased to 98% when pH = 3.

The CTS-coated cotton exhibits the ability in the separating immiscible oil-water mixtures due to its selective superwettability. The separation of the immiscible oil-water mixture is shown in Figure 6a. During separation, continuous water flowed through the CTS-coated cotton, and oil was rejected on the CTS-coated cotton. The separation efficiency and flux during the separation of various kinds of immiscible oil-water mixtures were also measured, as shown in Figure 6b. It is observed that the average separation efficiencies were higher than 99% for the separation of various immiscible oil-water mixtures and the separation flux was up to 42,000 L m^−2^ h^−1^, demonstrating the effectiveness and practicability in the separation of immiscible oil-water mixtures. In order to evaluate the stability of the CTS-coated cotton, the separating performance of the CTS-coated cotton in harsh environments was tested. Figure 6c illustrates the underwater oil contact angle and separation efficiency in aqueous environments with different pH values (from pH = 3 to pH = 11). It is observed that there were no obvious variation on underwater oil contact angles during variation of pH values, demonstrating the stability of the underwater superoleophobcity of the CTS-coated cotton. The oil-water separation efficiency was stable above 99% in the range of pH = 5 to pH = 11, whereas the separation efficiency decreased to 98% when pH = 3.

#### 3.3.2. Separation of Heavy Metal Ion-Contaminated Emulsions

**Figure 7:**
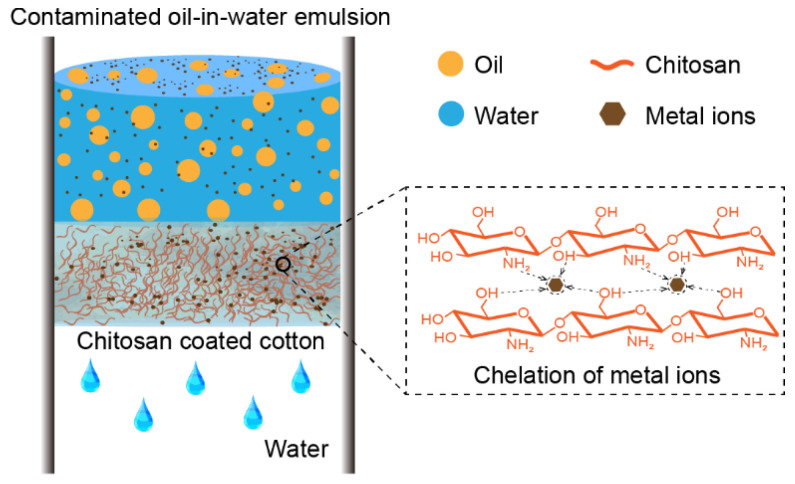
The mechanism of separation of heavy metal ion-contaminated oil-in-water emulsions with compressed CTS-coated cotton. The heavy metal ion-contaminated aqueous solution continuously flows through the cotton, during which the metal ions are absorbed by CTS, and the microdroplets of oil are rejected on the cotton surface due to the underwater superoleophobicity.

The oil-in-water emulsion is a more intensive type of oil-water mixture in which microdroplets of oil (normally smaller than 20 µm) are dispersed in water. High toxic heavy metal ion-contaminated aqueous solution widely exists in the emulsions which are created in industrial production. Benefit from the flexible compressibility of the substrate and the excellent chelation performance of CTS. The developed compressed CTS-coated cotton can not only separate stabilized oil-in-water emulsions but also remove various heavy metal ions dissolved in the aqueous solution of the emulsions during separation. The mechanism of this process is shown in Figure 7. The heavy metal ion-contaminated aqueous solution continuously flows through the CTS-coated cotton, during which the metal ions are absorbed by CTS due to the chelation interaction with the -NH_2_ and -OH groups. Meanwhile, the microdroplets of oil dispersed in the emulsion are rejected due to the underwater superoleophobicity of the CTS-coated cotton.

**Figure 8:**
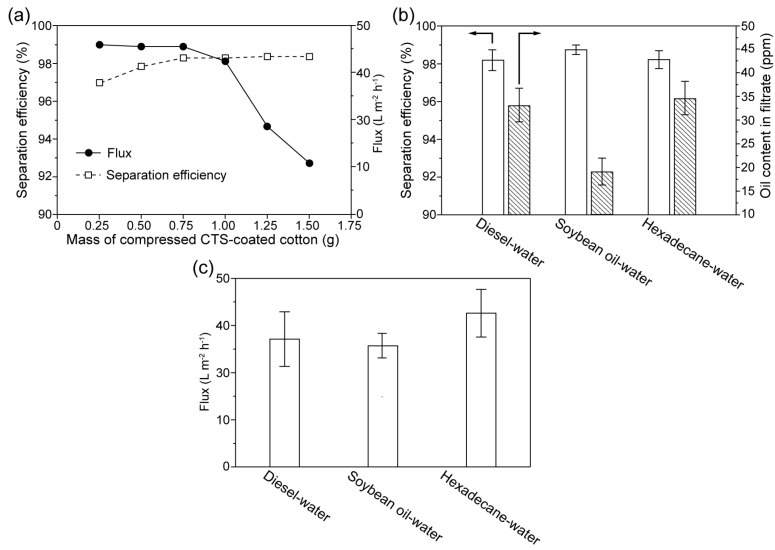
(**a**) The separation efficiency and the corresponding separation flux using CTS-coated cotton with constant compressed density (0.28 g cm^−3^) and different mass. (**b**) The efficiency in the separation of contaminated oil-in-water emulsions and the oil content in the corresponding filtrate using the optimized compressed CTS-coated cotton. The efficiencies were higher than 98% for separating various kinds of emulsions. (**c**) The fluxes during the separation of diesel-in-water, soybean oil-in-water and hexadecane-in-water emulsions. The flux varies from 30 L m^−2^ h^−1^ to 48 L m^−2^ h^−1^ for separating various kinds of emulsions.

To demonstrate the effect of separation and metal ion-removal capacity, various oil-in-water emulsions severely contaminated by Fe^3+^, Cu^2+^ and Cr^6+^, were selected as test samples. On the one hand, it is noted that the mass of coated CTS plays a key role in removing metal ions. In theory, the treated cotton with more CTS-coated fibers preserves higher metal ions removing efficiency. On the other hand, the emulsion separation efficiency and the separation flux depend on the compressed density and thickness of the CTS-coated cotton. Thus, in order to optimize the thickness of the compressed CTS-coated cotton, the separation efficiency and the corresponding separation flux using CTS-coated cotton with constant compressed density (0.28 g cm^−3^) and different mass was tested. As shown in Figure 8a, it is observed that the separation efficiency gradually increases with the increase of mass (thickness); the separation efficiency is stable when the mass of the CTS-coated cotton is larger than 0.75 g. The separation flux decreases with the increase of mass; this is because the increase of the cotton (thickness) enhances the resistance for liquid penetration. Thus, 1 g of the CTS-coated cotton with 0.28 g cm^−3^ of compressed density was selected as the optimization for emulsion separation. The separation efficiency and flux during separating different kinds of emulsions were also measured; the data is shown in Figure 8b,c. It can be observed that the optimized compressed CTS-coated cotton preserves a high efficiency over 98%, along with flux varying from 30 L m^−2^ h^−1^ to 48 L m^−2^ h^−1^ for separation various stabilized oil-in-water emulsions, which demonstrate the validity of the treated cotton in the emulsion separation applications.

**Figure 9:**
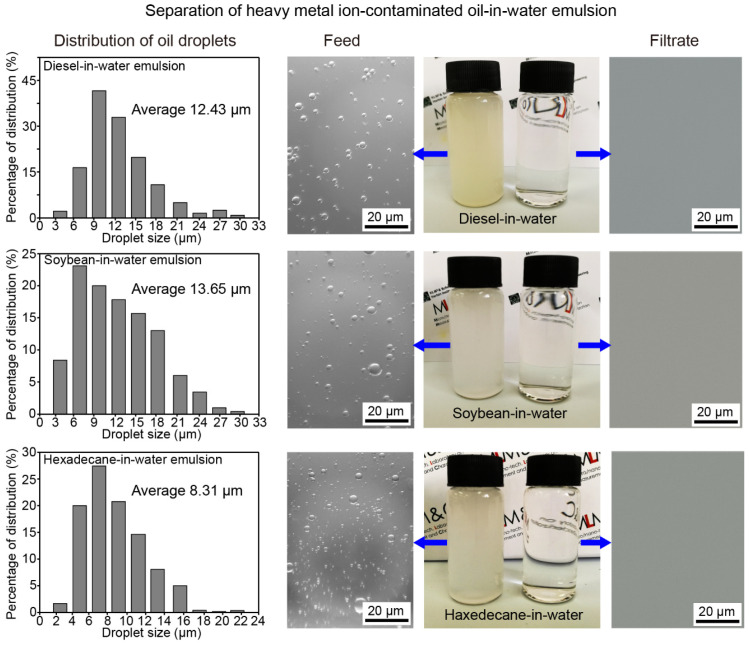
The effect of separation of contaminated diesel-in-water, soybean oil-in-water and hexadecane-in-water emulsions. Oil droplets which were smaller than 20 µm were detected in the feed before separation, whereas there were no droplets of oil detected in the filtrates.

The effect in the separation of oil-in-water emulsions was also evaluated, as shown in Figure 9. It is observed that all the test feeds were emulsified and milky, whereas the filtrates were transparent and clear. The separation effect was further demonstrated by the microscopic images before and after separation. The oil droplets whose diameters are smaller than 20 µm were detected in all the feed samples before separation, whereas there were no droplets of oil detected in the corresponding filtrates.

**Figure 10:**
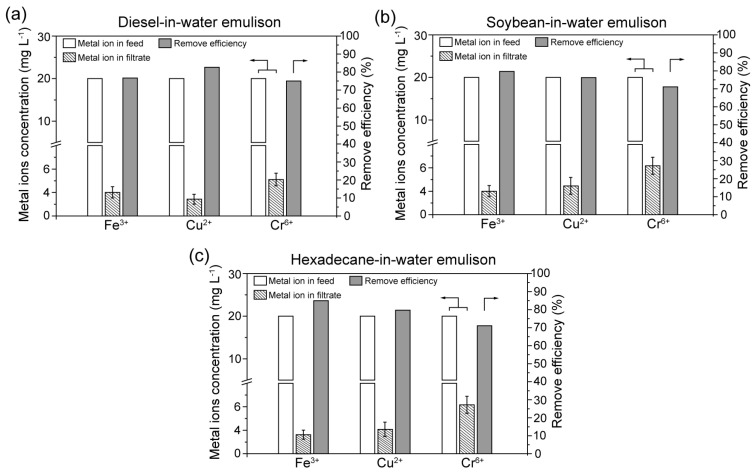
The concentration of heavy metal ions in the feed and filtrate and the corresponding removal efficiency during the separation of (**a**) contaminated diesel-in-water emulsion, (**b**) contaminated soybean oil-in-water emulsion and (**c**) contaminated hexadecane-in-water emulsion.

In order to evaluate the capacity in the removal of the heavy metal ions, the concentration of the remaining metal ion (Fe^3+^, Cu^2+^ and Cr^6+^) in the corresponding filtrate liquid after separation of the metal ion-contaminated oil-in-water emulsion was measured. The removal efficiency of the metal ion was calculated by Equation (3), and the data is exhibited in Figure 10. It is observed that the concentration of the metal ions remaining in all test filtrates experienced a noticeable decrease compared with the feed emulsions. The initial concentration of the metal ion pollutants in all the feed emulsions was 20 mg L^−1^, whereas the metal ion concentrations in the corresponding filtrates were no higher than 6.5 mg L^−1^. It is speculated that the relatively low separation flux endows the contaminated aqueous solution to have extended contact with the CTS, which leads to effective absorption of metal ions during penetration. The considerable removal efficiency which varies from 70% to 85% demonstrates effectiveness and practicability of the CTS-coated cotton in removing heavy metal ions.

## 4. Conclusions

In summary, a bioinspired eco-friendly, multi-functional chitosan-modified cotton was fabricated by coating chitosan solution on a cotton substrate. The treated cotton, which exhibits superhydrophilic and underwater superoleophobic properties, is non-toxic and can be used in the separation of both immiscible oil-water mixtures and oil-in-water emulsions due to the flexible compressibility of the cotton substrate. More significantly, by taking advantage of the chelate behavior between the chitosan molecule and the metal ions, it preserves the high-efficient ability of removing toxic heavy metal ions in an aqueous solution during the separation of emulsions. This eco-friendly CTS-coated cotton, which integrated both oil-water separation and purification capacities, demonstrates an attractive prospect for wastewater treatment in various applications.

## Figures and Tables

**Table 1:** Densities and surface tensions of the test liquids [1,23,25,44].

Liquid	Density (g cm^−3^)	Surface Tension ($\gamma_{L}$, mN m^−1^)
Hexadecane	0.77	27.05
Diesel	0.84	25.05
Soybean oil	0.92	45.50
Water	1.00	72.08
Diiodomethane	3.33	66.98
1,2-Dichloroethane	1.25	31.86

## Data Availability

Not applicable.
